# Development of a novel mouse model of hepatocellular carcinoma with nonalcoholic steatohepatitis using a high-fat, choline-deficient diet and intraperitoneal injection of diethylnitrosamine

**DOI:** 10.1186/s12876-016-0477-5

**Published:** 2016-06-13

**Authors:** Norihiro Kishida, Sachiko Matsuda, Osamu Itano, Masahiro Shinoda, Minoru Kitago, Hiroshi Yagi, Yuta Abe, Taizo Hibi, Yohei Masugi, Koichi Aiura, Michiie Sakamoto, Yuko Kitagawa

**Affiliations:** Department of Surgery, School of Medicine, Keio University, 35 Shinanomachi, Shinjuku-ku, Tokyo, 160-8582 Japan; Chugai Pharmaceutical Endowed Research Chair in Molecular Targeted Therapy of Gastrointestinal Cancer, School of Medicine, Keio University, Tokyo, Japan; Department of Pathology, School of Medicine, Keio University, Tokyo, Japan; Department of Surgery, Kawasaki Municipal Hospital, Kawasaki-ku, Japan

**Keywords:** Nonalcoholic steatohepatitis, Hepatocellular carcinoma, Diethylnitrosamine, High-fat choline-deficient diet, Mouse model

## Abstract

**Background:**

The incidence of hepatocellular carcinoma with nonalcoholic steatohepatitis is increasing, and its clinicopathological features are well established. Several animal models of nonalcoholic steatohepatitis have been developed to facilitate its study; however, few fully recapitulate all its clinical features, which include insulin resistance, inflammation, fibrosis, and carcinogenesis. Moreover, these models require a relatively long time to produce hepatocellular carcinoma reliably. The aim of this study was to develop a mouse model of hepatocellular carcinoma with nonalcoholic steatohepatitis that develops quickly and reflects all clinically relevant features.

**Methods:**

Three-week-old C57BL/6J male mice were fed either a standard diet (MF) or a choline-deficient, high-fat diet (HFCD). The mice in the MF + diethylnitrosamine (DEN) and HFCD + DEN groups received a one-time intraperitoneal injection of DEN at the start of the respective feeding protocols.

**Results:**

The mice in the HFCD and HFCD + DEN groups developed obesity early in the experiment and insulin resistance after 12 weeks. Triglyceride levels peaked at 8 weeks for all four groups and decreased thereafter. Alanine aminotransferase levels increased every 4 weeks, with the HFCD and HFCD + DEN groups showing remarkably high levels; the HFCD + DEN group presented the highest incidence of nonalcoholic steatohepatitis. The levels of fibrosis and steatosis varied, but they tended to increase every 4 weeks in the HFCD and HFCD + DEN groups. Computed tomography scans indicated that all the HFCD + DEN mice developed hepatic tumors from 20 weeks, some of which were glutamine synthetase-positive.

**Conclusions:**

The nonalcoholic steatohepatitis-hepatocellular carcinoma model we describe here is simple to establish, results in rapid tumor formation, and recapitulates most of the key features of nonalcoholic steatohepatitis. It could therefore facilitate further studies of the development, oncogenic potential, diagnosis, and treatment of this condition.

**Electronic supplementary material:**

The online version of this article (doi:10.1186/s12876-016-0477-5) contains supplementary material, which is available to authorized users.

## Background

Worldwide, hepatocellular carcinoma (HCC) is the fifth-most common cancer in men and seventh-most common in women, and its incidence has continuously increased in recent years [1]. The major risk factors for HCC are infection with hepatitis B and C viruses; however, the incidence of viral-related HCC has decreased owing to improvements in the management and treatment of viral infections. Meanwhile, the frequency of non-viral HCC—related to alcohol consumption and other factors—has gradually increased. Nonalcoholic fatty liver disease (NAFLD), which is a hepatic manifestation of metabolic syndrome, is one of the most common causes of chronic liver disease and liver cirrhosis in the world [2, 3]. NAFLD ranges from simple steatosis to nonalcoholic steatohepatitis (NASH) associated with inflammation, fibrosis, and carcinogenesis [4]. In accordance with multiple-hit theory, metabolic syndrome, genetic factors, oxidative stress, inflammatory cytokines, endotoxins, and insulin resistance have been shown to be involved in NASH development and the progression of NASH-HCC [5]. A number of studies describing the natural history of NASH have found liver failure and HCC to be the major causes of death [6–12].

Various genetic and dietary NASH animal models exist. For example, *PTEN* knockout mice undergo carcinogenesis, and exhibit steatohepatitis, but not obesity, dyslipidemia, or insulin resistance [13]. *ob*/*ob* mice are diabetic owing to a defect in the *leptin* gene and genetically obese; *db*/*db* mice have a defective leptin receptor gene [14, 15]. Dietary models include a high-fat diet (HFD) model [16], a high-fat, choline-deficient diet model (HFCD) [17, 18], a methionine- and choline-deficient diet (MCD) model [19, 20]. These models require a relatively long period—usually about 1 year—to produce HCC [17]. A 16-week NASH-HCC mouse model based on an HFD combined with low-dose streptozotocin (STZ) has been reported [21]; however, those mice were not insulin resistant, because they exhibited a lack of insulin secretion. The liver carcinogenicity of diethylnitrosamine (DEN) has been reported [22–24], and DEN has been added to the rat NASH-HCC model in combination with an HFD [25–27]. A few models exhibit all the associated clinical features of NASH-HCC, such as insulin resistance, inflammation, fibrosis, and carcinogenesis, such as a high-fat and fructose diet model [28]. Recent genetic and dietary NASH-HCC models have included MUP-uPA transgenic mice with HFD [29] and melanocortin 4 receptor (MC4R) knockout mice with HFD [30].

Since there is no effective treatment or chemoprevention for HCC related to NASH, a mouse model with the same clinical features as human NASH is needed. In this study, by feeding C57BL/6 mice an HFCD combined with DEN exposure, we developed a novel experimental NASH-HCC mouse model that exhibits all the relevant clinical features by 20 weeks, including insulin resistance, inflammation, fibrosis, and carcinogenesis.

## Methods

### Animals

**Fig. 1:**
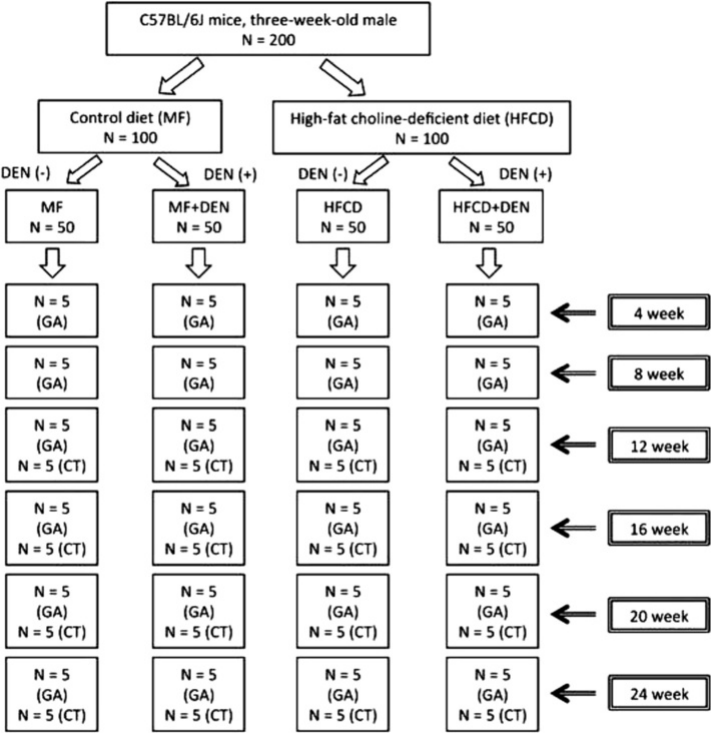
Overview of the experimental design, showing intraperitoneal diethylnitrosamine (DEN) administration. N, number; GA, general assessment; CT, computed tomography scanning

Three-week-old male C57Bl/6J mice were purchased from Oriental Yeast (Tokyo, Japan), housed in a temperature-, humidity-, and ventilation-controlled vivarium, and kept on a 12-h light/dark cycle under specific pathogen-free conditions. For the DEN intraperitoneal (i.p.) experiment, the mice were randomly divided into two groups: the standard diet (MF) group, which was fed an MF (11.4 % fat, 25.7 % protein, 62.9 % carbohydrate, total calories 359 kcal/100 g; purchased from Oriental Yeast); and the HFCD group, which was fed an HFCD (58.0 % fat, 16.4 % protein, 25.5 % carbohydrate, total calories 556 kcal/100 g; purchased from Oriental Yeast) [17]. The two groups were further divided into two subgroups, one of which was treated with DEN. The MF + DEN and HFCD + DEN subgroups received a one-time i.p. injection of 25 mg/kg DEN at the start of the respective feeding protocols. Food and water were given ad libitum. Five mice from each group were sacrificed every 4 weeks, and their body weights and liver weights measured. An overview of the experimental protocol appears in Fig. 1.

All procedures for animal experimentation were in accordance with the Helsinki Declaration of 1975 and Institutional Guidelines on Animal Experimentation at Keio University. This study was approved by the Keio University Institutional Animal Care and Use Committee (Approval number: 08073).

### Measurement of biological parameters

Serum levels of fasting blood sugar (FBS), alanine aminotransferase (ALT), and triglyceride (TG) were measured using a Fuji Dri-Chem 3500 analyzer (Fuji Film Co. Ltd, Tokyo, Japan). Insulin levels were determined using a mouse insulin enzyme-linked immunoassay kit (Morinaga Institute of Biological Science, Inc. Yokohama, Japan). The quantitative insulin sensitivity check index was calculated as 1/log (fasting insulin) + log (fasting glucose). Interleukin (IL)-6, tumor necrosis factor (TNF)-alpha, leptin, adiponectin, and C-reactive protein (CRP) levels were measured using Procarta Multiplex Immunoassays (Affymetrix eBioscience, San Diego, CA, USA). Serum amyloid A (SAA) was determined using the PHASE RANGE Mouse SAA ELISA kit (Tridelta Development Ltd., Kildare, Ireland).

### Insulin tolerance test

To assess insulin resistance, we performed an insulin tolerance test. Mice were injected with 1 U/kg of insulin (Humulin R; Eli Lilly Japan, Kobe, Japan), and blood glucose was measured using an Accu-Chek meter (Roche Diagnostics Japan, Tokyo, Japan) every 20 min up to 120 min. The ratio was calculated using the pre-injection value as a standard.

### Histological analysis

Liver tissue was assessed grossly, and samples were fixed in 10 % formaldehyde and processed for hematoxylin-eosin staining. Variables were blindly scored by two experienced hepatopathologists using a modified scoring system adapted from the NAFLD activity score (NAS): macrosteatosis (0–3); lobular inflammatory changes (0–3); hepatocyte ballooning (0–2); and fibrosis scored as portal and perivenular (stage 0–4).

Immunohistochemical detection of F4/80 (a macrophage marker) was performed as follows. Paraffin sections were deparaffinized in xylene, hydrated in a gradient of ethanol, and incubated with proteinase K for 10 min at room temperature for antigen retrieval. The sections were then incubated with a primary rat anti-mouse F4/80 antibody (T-2006; BMA Biomedicals, Augst, Switzerland) overnight at 4 °C, followed by incubation with Histofine Simple Stain mouse MAX-PO (Rat) (Nichirei Bioscience, Tokyo, Japan) for 30 min. Staining was detected using diaminobenzidine tetrahydrochloride. Three light microscopy images at 100 times magnification were taken of each slide to determine the ratio of F4/80-positive cells (macrophages) to hepatocyte nuclei.

Immunostaining for glutamine synthetase (GS) was performed by the Stelic Institute & Co., Inc. (Tokyo, Japan). Sirius red staining was performed using Van Gieson’s stain solution and Sirius red solution from Muto Pure Chemicals Co., Ltd. (Tokyo, Japan). The degree of liver fibrosis was assessed using Histoquest (Tissue Gnostics, Vienna, Austria) using the three images of each slide described above. The caudate liver lobes were embedded in Tissue-Tek OCT compound (Sakura Finetechnical Co., Ltd., Tokyo, Japan) and snap-frozen in liquid nitrogen. Frozen sections, 5 μm thick, were fixed with 50 % ethanol for 5 min and stained with Sudan III (Wako Pure Chemical Industries Ltd., Osaka, Japan) in 55 % ethanol for 1.5 h at room temperature.

### X-ray computed tomography

For the detection and characterization of tumor development, the mice were imaged using the in vivo three-dimensional micro X-ray computed tomography (CT) system R-mCT2 (Rigaku, Tokyo, Japan). The X-ray tube voltage, current, and field of view were 90 kV, 200 μA, and 30 mm, respectively. ExiTron nano 6000 (Miltenyi Biotec, Bergisch Gladbach, Germany) was injected into the tail vein of the mice on the day before the CT scan at a dose of 10 mL/kg. ExiTron nano 6000 is uptake by Kupffer cells in liver, therefore, we defined the nodules which were not enhanced by ExiTron nano 6000. Using 512 CT image of samples, largest diameter of node and numbers were measured by Osirix software (OnDemand software, Cybermed Inc., Bernex, Switzerland).

### Comparison of HFCD + DEN and HFD32 + DEN

High Fat Diet 32 (HFD32) was obtained from CLEA Japan, Inc. (Tokyo, Japan). This diet consists of 32.0 % fat, 25.5 % protein, 29.4 % carbohydrate, and total calories of 507.6 kcal/100 g. Five 3-week-old male mice were fed HFD32 and received a one-time i.p. injection of 25 mg/kg DEN at the start of the feeding protocol. After 4 weeks, liver tissue was taken and snap-frozen in liquid nitrogen.

RNA was extracted from frozen liver tissue after 4 weeks of MF, HFCD + DEN, and HFD32 + DEN using the RNeasy Mini Kit (Qiagen, Hilden, Germany). Genome-wide mRNA expression levels were determined using the Superprint G3 Mouse GE microarray kit 8 x 60 k Ver 2.0, which contains 27,122 genes (G4858A#074809, Agilent Technologies, South Queensferry, UK). All microarray data of the HFCD + DEN mice and HFD32 + DEN mice were normalized with data of the MF mice using GeneSpring GX Ver 13.1 software (Agilent Technologies); the threshold was set at more than twofold changes. We analyzed the data by means of Qiagen’s Ingenuity Pathway Analysis (IPA) software Ver 1.0 (Qiagen, Hilden, Germany) for functional analysis. Molecules from the dataset that exceeded the twofold cutoff and were associated with biological function or diseases in the Ingenuity Knowledge Base were considered for analysis. The right-tailed Fisher’s exact test was used to calculate a *p* value to determine the probability that each biological function or disease assigned to that dataset was due to chance alone.

### Statistical analysis

The data are shown as the mean ± standard deviation or number (%). The Mann–Whitney *U* test was used for the analysis of body and liver weight and ALT, TG, and leptin levels. We performed all statistical analyses using IBM SPSS Statistics 21 software (SPSS, Inc., Chicago, IL, USA).

## Results

### Body weight, liver weight, and laboratory findings

**Fig. 2:**
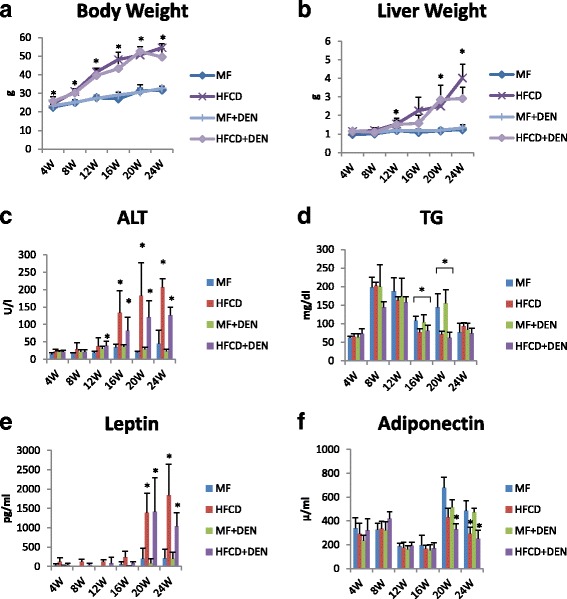
Body and liver weights and laboratory findings: **a** body weight; **b** liver weight; **c** plasma alanine aminotransferase (ALT); **d** plasma triglycerides (TG); **e** plasma leptin; and **f** adiponectin. The data are shown as the mean + standard deviation. * *p* < 0.05 indicates a significant difference between the standard diet (MF) group and the other groups for each month. HFCD, high-fat choline-deficient diet; DEN, diethylnitrosamine

To develop the NASH-HCC model, we used a combination of an HFCD and i.p. DEN administration. The mice were divided into four groups: MF; HFCD; MF + DEN; and HFCD + DEN. Animals in the MF + DEN and HFCD + DEN groups received a single i.p. injection of DEN at the start of the respective feeding protocols. At 24 weeks, the mean body weights of the MF, HFCD, MF + DEN, and HFCD + DEN mice were 31.7 g, 54.5 g, 32.6 g, and 49.5 g (Fig. 2a), respectively; the mean liver weights were 1.2 g, 4.0 g, 1.3 g, and 2.9 g (Fig. 2b), respectively. Both body weight and liver weight were significantly higher in the HFCD and HFCD + DEN groups than in the MF and MF + DEN groups.

Plasma ALT levels increased every 4 weeks, with the HFCD and HFCD + DEN groups showing remarkably high levels (Fig. 2c). Plasma TG levels peaked at 8 weeks for all four groups and decreased thereafter (Fig. 2d). There were significant differences in TG levels between the MF and HFCD + DEN groups at 16 and 20 weeks. Plasma leptin levels increased from 20 weeks in the HFCD and HFCD + DEN groups (Fig. 2e). Plasma adiponectin levels decreased from 20 weeks in the HFCD and HFCD + DEN groups (Fig. 2f).

The levels of other biomarkers, such as FBS, CRP, IL-6, and TNF-alpha, are shown in Table 1. CRP levels increased from 20 weeks in the HFCD and HFCD + DEN groups, though there was no significant difference. However, compared with the MF group, serum levels of TNF-alpha were higher in the HFCD group at 4 and 8 weeks and in the HFCD + DEN group at 8 weeks. Serum levels of IL-6 tended to be higher in the HFCD and HFCD + DEN groups; however, at 16 weeks, only the HFCD group exhibited significantly different levels compared with the MF group.

**Table 1:** Summary of laboratory findings for FBS, CRP, IL-6, TNF-alpha, and adiponectin levels

	Week	MF	HFCD	MF + DEN	HFCD + DEN
FBS (mg/dl)	4	211.3 ± 41.7	297.5 ± 24.6	191.0 ± 11.2	282.3 ± 26.5
	8	149.6 ± 30.9	174.2 ± 32.9	177.0 ± 64.9	167.6 ± 31.3
	12	167.6 ± 41.6	241.6 ± 32.4	152.0 ± 24.2	273.4 ± 31.5
	16	109.8 ± 11.4	245.0 ± 46.4	112.8 ± 8.7	189.8 ± 52.5
	20	188.4 ± 14.6	263.2 ± 36.0	172.0 ± 36.7	242.0 ± 14.4
	24	165.2 ± 23.9	265.2 ± 6.5	205.3 ± 32.5	249.4 ± 24.2
CRP (ng/ml)	4	4552.6 ± 2533.3	3756.8 ± 1366.4	4284.9 ± 2261.0	4915.4 ± 1697.0
	8	5803.1 ± 1368.7	13324.9 ± 7100.6	5837.7 ± 2422.0	6568.3 ± 3827.0
	12	2412.0 ± 211.8	2505.3 ± 517.7	2465.9 ± 170.5	2427.1 ± 337.3
	16	2080.4 ± 756.5	3065.7 ± 1047.4	2801.3 ± 457.0	3002.1 ± 599.5
	20	2125.1 ± 306.9	3170.3 ± 711.8	2185.4 ± 256.3	3293.4 ± 416.6
	24	2227.6 ± 613.8	3143.3 ± 393.3	2044.0 ± 349.9	3686,7 ± 481.2
IL-6 (pg/ml)	4	0.2 ± 0.3	1.3 ± 1.5	0.3 ± 0.5	0.9 ± 1.3
	8	0.5 ± 0.5	1.8 ± 1.5	0.6 ± 0.7	0.9 ± 1.2
	12	0.8 ± 0.5	1.1 ± 0.7	1.1 ± 0.8	2.0 ± 1.5
	16	1.3 ± 0.6	2.9 ± 1.2	1.2 ± 0.8	0.9 ± 0.2
	20	1.6 ± 1.2	1.3 ± 0.8	1.4 ± 0.9	1.6 ± 0.9
	24	2.1 ± 1.3	4.2 ± 1.8	2.2 ± 4.5	1.6 ± 0.3
TNF-alpha (pg/ml)	4	N.D.	20.7 ± 28.9	N.D.	6.2 ± 12.0
	8	1.8 ± 3.9	270.9 ± 297.5	5.0 ± 7.1	134.6 ± 153.7
	12	2.2 ± 2.8	0.4 ± 0.9	0.9 ± 2.0	1.9 ± 3.1
	16	0.1 ± 0.1	3.4 ± 4.8	0.1 ± 0.3	N.D.
	20	1.2 ± 1.6	2.0 ± 2.2	1.4 ± 1.4	1.4 ± 1.3
	24	3.8 ± 6.4	5.8 ± 3.0	3.5 ± 5.2	0.5 ± 1.2

The data are shown as the mean ± standard deviation. Each group contained five mice. *FBS* fasting blood sugar, *CRP* C-reactive protein, *IL* interleukin, *TNF*-alpha tumor necrosis factor-alpha

### Insulin resistance

**Fig. 3:**
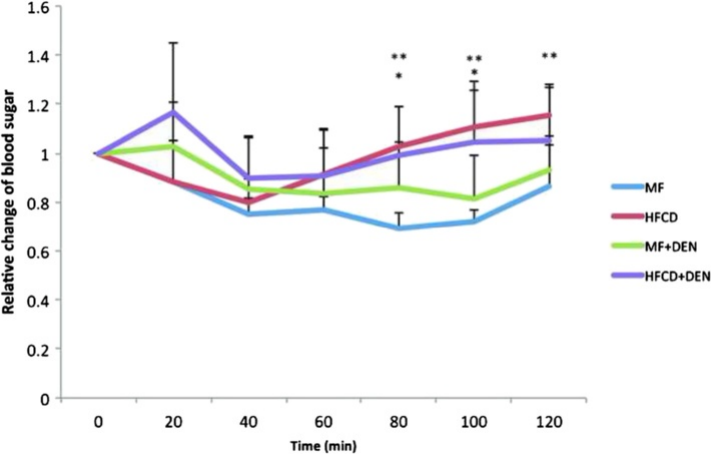
Insulin tolerance test at 12 weeks. The data appear as the mean + standard deviation. * *p* < 0.05 indicates a significant difference between the standard diet (MF) group and the high-fat, choline-deficient diet (HFCD) + diethylnitrosamine (DEN) group. ** *p* < 0.05 indicates a significant difference between the MF and HFCD groups

Insulin resistance was calculated using the quantitative insulin sensitivity check index. All mice in the HFCD and HFCD + DEN groups had developed insulin resistance at 12 weeks, whereas animals in the MF and MF + DEN groups had developed insulin resistance at 24 weeks (Table 2). To confirm the development of insulin resistance, we performed an insulin tolerance test. There was a significant difference in insulin resistance between the MF and HFCD + DEN groups at 80 and 100 min. There was also a significant difference in insulin resistance between the MF and HFCD groups at 80, 100, and 120 min (Fig. 3).

**Table 2:** Insulin resistance calculated using the quantitative insulin sensitivity check index

	4 W	8 W	12 W	16 W	20 W	24 W
MF	N.D.	**0.227**	**0.139**	N.D.	N.D.	0.413
	**0.064**	N.D.	0.348	N.D.	0.445	**0.12**
	N.D.	0.381	**0.193**	N.D.	N.D.	**0.249**
	N.D.	N.D.	0.416	0.315	N.D.	0.777
	N.D.	N.D.	0.672	N.D.	N.D.	**0.083**
HFCD	N.D.	**0.22**	**0.089**	**0.058**	**0.026**	**0.045**
	**0.2002**	**0.206**	**0.047**	**0.034**	**0.026**	**0.025**
	0.3954	**0.168**	**0.067**	**0.034**	**0.057**	**0.025**
	**0.0705**	N.D.	**0.081**	**0.027**	**0.034**	**0.018**
	**0.1384**	N.D.	**0.046**	**0.079**	**0.037**	**0.030**
MF + DEN	1.047	N.D.	0.579	N.D.	N.D.	**0.2916**
	N.D.	N.D.	N.D.	N.D.	N.D.	**0.116**
	N.D.	0.916	N.D.	0.642	N.D.	**0.131**
	N.D.	0.916	N.D.	N.D.	N.D.	**0.153**
	N.D.	0.159	N.D.	9.101	**0.245**	N.D.
HFCD + DEN	1.047	**0.0808**	**0.016**	**0.072**	**0.033**	**0.026**
	N.D.	**0.1167**	**0.031**	**0.054**	**0.063**	**0.020**
	**0.1026**	0.377	**0.030**	**0.025**	**0.069**	**0.034**
	**0.0826**	0.315	**0.048**	0.314	**0.201**	**0.031**
	**0.172**	**0.173**	**0.036**	**0.022**	**0.055**	**0.037**

The quantitative insulin sensitivity check index = 1/log (fasting insulin) + log (fasting glucose). A value < 0.3 indicates insulin resistance; values from 0.348 to 0.430 are normal, and values ≥ 3.0 indicate high insulin sensitivity (type 1 diabetes mellitus). Index values < 0.3 (insulin resistance) are in bold. Each group contained five mice. *W* weeks, *MF* standard diet, *HFCD* high-fat choline-deficient diet, *DEN* diethylnitrosamine, *N.D* not determined

### Histological findings of non-tumor tissue

**Fig. 4:**
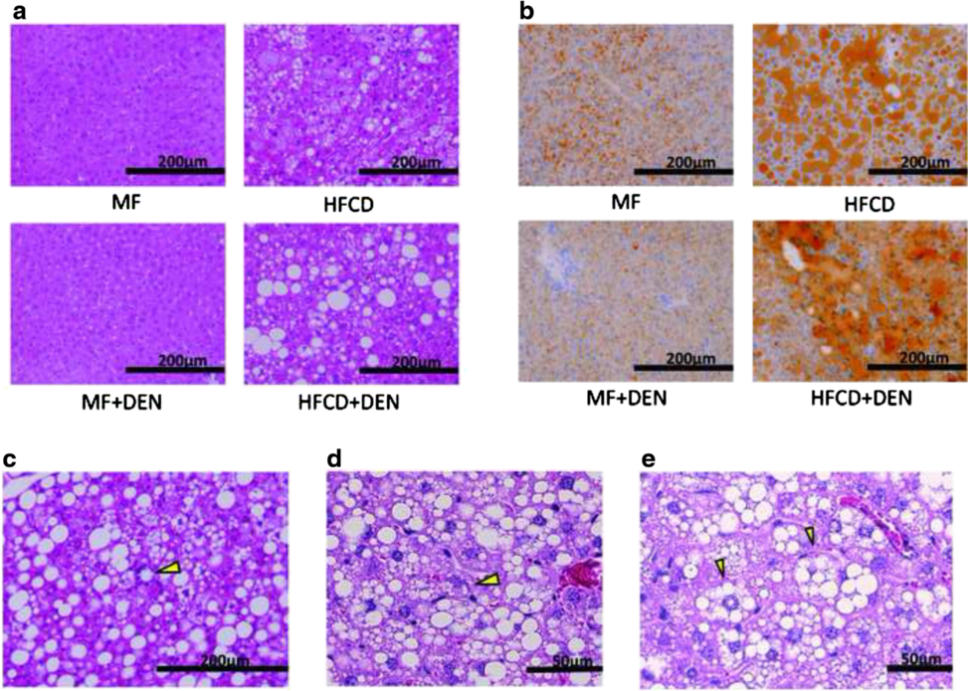
Representative images of stained liver sections: **a** 12 weeks with hematoxylin-eosin staining; **b** 12 weeks with Sudan staining; **c**–**e** 16 weeks in HFCD + DEN mice with hematoxylin-eosin staining. The original magnification is × 200 (**a**–**c**) and × 400 (**d**). Lipogranuloma (**c**), a Mallory-Denk body (**d**), and hepatocyte ballooning (**e**) are indicated by yellow arrowheads. MF, standard diet; HFCD, high-fat choline-deficient diet; DEN, diethylnitrosamine

Liver specimens were evaluated using hematoxylin-eosin staining. At 12 weeks, mice in the HFCD and HFCD + DEN groups evidenced fat accumulation, lobular inflammation, and hepatocyte ballooning, which are characteristic of NASH. These changes were more evident in specimens from the HFCD + DEN group than in those from the HFCD group (Fig. 4a). We observed no apparent pathological findings, including fatty degeneration or necroinflammatory changes in hepatocytes in the hematoxylin–eosin-stained tissue of MF or MF + DEN mice. Sudan III staining revealed remarkable macrovesicular fat accumulation in both the HFCD and HFCD + DEN groups at 12 weeks; microvesicular fat accumulation was evident in the MF group—and to a lesser extent in the MF + DEN group (Fig. 4b). Lipogranuloma (Fig. 4c), Mallory-Denk bodies (Fig. 4d), and hepatocyte ballooning (Fig. 4e), which are characteristic of NASH, were observed in the HFCD + DEN mice from 16 weeks after feeding.

NAS is an established scoring system for assessing the severity of NASH. In the NAS system, a score of 3–5 represents possible or borderline NASH; a score greater than 5 indicates definite NASH. The NAS was possible or borderline from 16 weeks and definite from 20 weeks in the HFCD mice; it was possible or borderline from 12 weeks and definite from 16 weeks in the HFCD + DEN group (Table 3).

**Table 3:** Nonalcoholic fatty liver disease activity score (NAS)

	Week	MF	HFCD	MF + DEN	HFCD + DEN
NAS	4	0	0.6 ± 0.9	0.6 ± 0.6	1.3 ± 0.5
	8	0	0.8 ± 0.8	0.4 ± 0.6	1.2 ± 1.6
	12	0	2.6 ± 1.7	1 ± 0	3 ± 2.0
	16	0.2 ± 0.5	4.6 ± 2.2	1 ± 0	5.2 ± 1.3
	20	0	5.2 ± 0.5	1 ± 0	5.4 ± 0.6
	24	0	6.2 ± 0.8	1 ± 0	5.6 ± 0.6

Each group contained five mice. *MF* standard diet, *HFCD* high-fat choline-deficient diet, *DEN* diethylnitrosamine

**Fig. 5:**
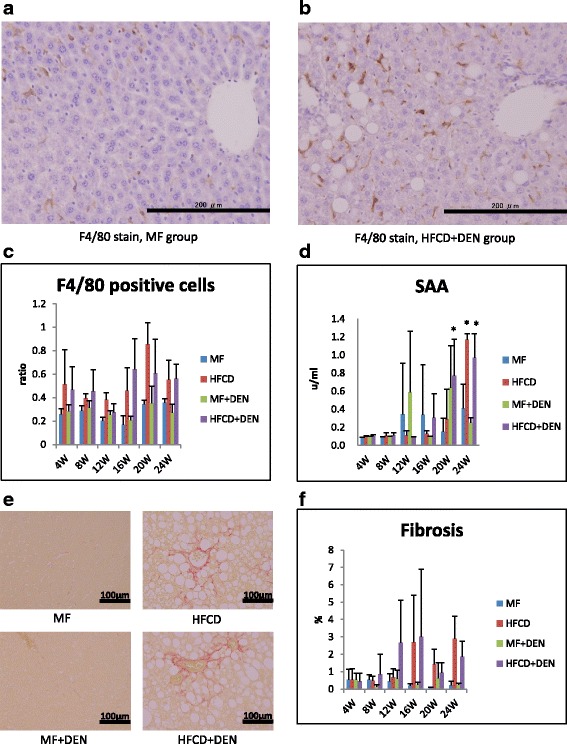
Immunohistochemistry with F4/80 antibody (×200 original magnification) at 4 weeks: **a** standard diet (MF) group; **b** high-fat, choline-deficient diet (HFCD) + diethylnitrosamine (DEN) group; **c** the ratio of F4/80-positive cells (macrophages) to hepatocyte nuclei; **d** serum amyloid A (SAA) immunostaining; **e** representative images of liver sections stained with Sirius red at 24 weeks; and **f** proportion of fibrotic area measured using Histoquest. The data are shown as the mean + standard deviation

To evaluate inflammation, we undertook immunohistochemical detection of macrophages with F4/80 antibody and SAA measurement. Representative images of macrophages in the perivenular zone at 4 weeks in the MF and HFCD + DEN mice are presented in Fig. 5a, b. The ratio of F4/80-positive cells (macrophages) to hepatocyte nuclei was higher in the HFCD and HFCD + DEN groups than in the other two groups from 4 weeks (Fig. 5c). SAA was significantly higher after 20 weeks in the HFCD + DEN mice and at 24 weeks in the HFCD mice (Fig. 5d). Fibrosis was more conspicuous in the HFCD and HFCD + DEN mice than in the other two groups (Fig. 5e). The area of fibrosis increased dramatically from 12 weeks in the HFCD + DEN mice and from 16 weeks in the HFCD group (Fig. 5f).

### CT scans and immunohistochemistry of hepatic tumors

**Fig. 6:**
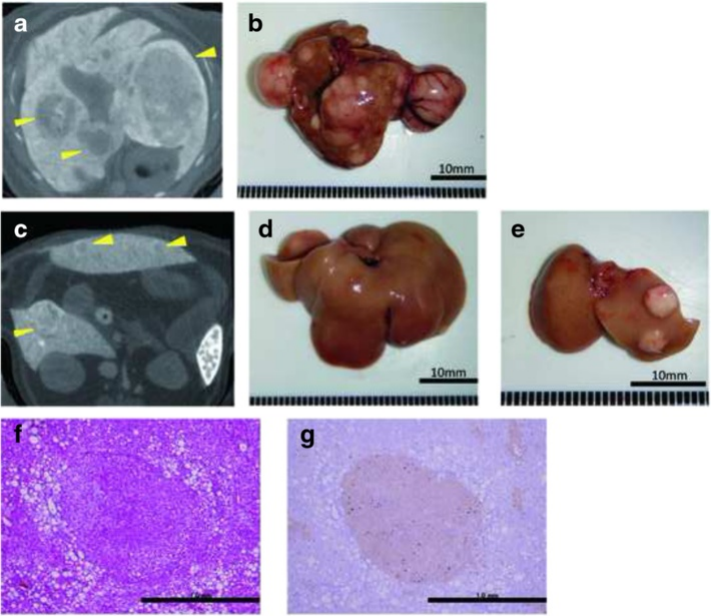
Computed tomography scans and immunohistochemistry of hepatic tumors in the high-fat, choline-deficient (HFCD) + diethylnitrosamine (DEN) group at 24 weeks: **a**, **c** computed tomography findings; **b**–**e** macroscopic views. The image in **a** is a section of the whole liver depicted in **b**; the image in **c** is a section of the whole liver shown in **d**; and the image in panel **e** depicts the right and left medial lobes of the whole liver in panel **d**. The lesions are indicated by yellow arrowheads. **f** Hematoxylin-eosin staining of the liver tumor. **g** Immunohistochemical staining for glutamine synthetase

To evaluate tumor development, we performed a CT scan every 4 weeks from week 12. The largest liver mass had a maximum diameter of 13 mm at 24 weeks in the HFCD + DEN group (Fig. 6a, b). Small nodules were typically seen in the liver macroscopically and by CT scan at 24 weeks (Fig. 6c–e). Positive findings were evident in 20 % and 100 % of the HFCD + DEN mice at 16 weeks and 20 weeks, respectively. Only one mouse had positive findings in the HFCD group at 20 weeks and one mouse in the MF + DEN group at 24 weeks. In the HFCD + DEN group, there were on average eight tumors at 24 weeks, with an average size of 2.9 mm (Table 4). To confirm malignancy, we immunostained the tumors to detect GS. GS-positive HCC was found in some specimens (Fig. 6f, g), although not all tumors were stained.

**Table 4:** Summary of computed tomography findings, rate of positive findings, tumor number, and tumor size from 12 to 24 weeks

	Week	MF	HFCD	MF + DEN	HFCD + DEN
Mice with positive findings, n (%)	12	0	0	0	0
	16	0	0	0	1/5 (20 %)
	20	0	1/5 (20 %)	0	**5/5 (100 %)**
	24	0	0	1/5 (20 %)	**5/5 (100 %)**
Tumor number	12	0	0	0	0
	16	0	0	0	5
	20	0	6	0	10.0 ± 5.4
	24	0	0	1	8.8 ± 5.4
Tumor size (mm)	12				
	16				0.6 ± 0.4
	20		0.8 ± 0.2		1.6 ± 0.8
	24			2.2	2.9 ± 2.8

Data are shown as the mean ± standard deviation. Data in bold indicate a significant difference (*p* < 0.05) between the standard diet (MF) group and the other groups for each month. *HFCD* high-fat choline-deficient diet, *DEN* diethylnitrosamine

### Comparison of HFCD + DEN and HFD32 + DEN

**Fig. 7:**
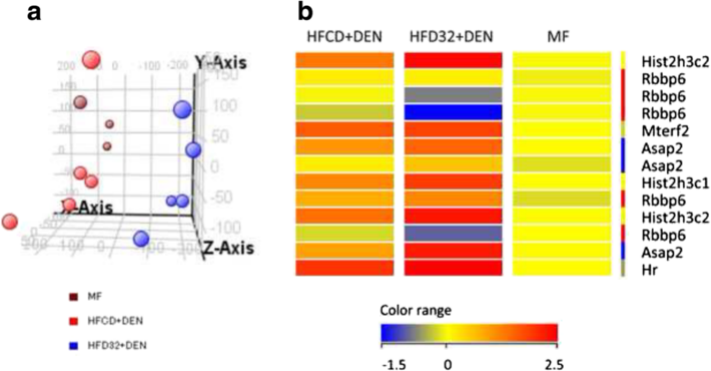
Expression of MF, HFCD + DEN, and HFD32. **a** Principal component analysis of MF (brawn), HFCD + DEN (red), and HFD32 + DEN (blue). MF group contained three mice. HFCD + DEN and HFD32 + DEN group contained five mice. **b** Heat map of HCC-related genes common to both HFCD + DEN and HFD32 + DEN. DEN, diethylnitrosamine; standard diet (MF); HFCD, high-fat, choline-deficient diet; HFD32, high-fat diet

To determine why HFCD + DEN promoted cancer development, we performed RNA microarray analysis. For this, we used HFD32, which is a widely employed high-fat diet. Principal component analysis provides a way of identifying predominant gene expression patterns. Surprisingly, the general expression of HFCD + DEN was closer to MF than to HFD32 + DEN (Fig. 7a). Clustering analysis and gene ontology analysis indicated that probably as a result of hepatitis, HFCD + DEN and HFD32 commonly changed the expression gene related to defense response and immune response. Functional analysis extracted 13 genes from HFCD + DEN and 163 from HFD32 + DEN related to HCC: HFCD + DEN and HFD32 + DEN were found to have six genes in common. As seen in the heat map in Fig. 7b and Additional file 1, expression of Histone cluster 1, H3c (Hist1h3c), histone cluster 1, H3g (Hist1h3g), Mitochondrial transcription termination factor 2 (Mterf2), ArfGAP with SH3 domain, ankyrin repeat and PH domain 2 (Asap2), and Hair growth associated (Hr) showed an increase in both the HFCD + DEN and HFD32 + DEN groups. Expression of Retinoblastoma binding protein 6 (Rbbp6) in HFCD + DEN presented a slight decrease, though it was a large decrease in HFD32 + DEN.

## Discussion

**Fig. 8:**
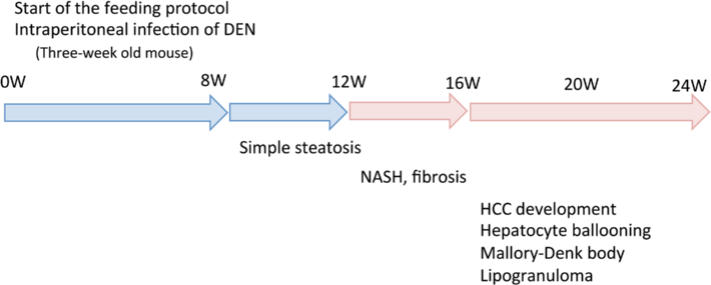
Summary of the experimental model. DEN, diethylnitrosamine; HCC, hepatocellular carcinoma; NASH, nonalcoholic steatohepatitis

It has been reported that the development and progression of NASH-HCC follows a multiple-hit pathway, which includes metabolic syndrome, genetic factors, oxidative stress, inflammatory cytokine release, endotoxins, and insulin resistance [5]. Previous NASH models have combined two or more of these hits by using a special diet with a chemical agent [21, 27] or specific genetic changes [14, 20]. However, these demand relatively long periods before the onset of HCC. NASH models based only on an HFCD require considerably longer periods—usually more than 1 year—to reliably produce carcinoma [17]. By combining an HFCD with a chemical agent, DEN, our model resulted in carcinoma within 20 weeks. DEN increases oxidative stress [31], which is one of the most important factors in the development and progression of NASH since it stimulates Kupffer cells [32]. Mice in the HFCD + DEN group showed elevated SAA levels, a higher NAS, and earlier fibrosis than those in the HFCD group. We also demonstrated that our NASH mouse model—based on an HFCD combined with i.p. injection of DEN—stimulated insulin resistance, fibrosis, and HCC within 20 weeks (Fig. 8).

The MCD model is one of the best-known NASH animal models [19, 20]. Choline deficiency causes Cyp2E1 upregulation with increased reactive oxygen species formation, lipid peroxidation, and mitochondrial dysfunction [27]; methionine deficiency exacerbates hepatic injury associated with oxidative and endoplasmic reticulum stress [33]. Although MCD mice develop steatohepatitis, fibrosis, and carcinogenesis, both body weight and insulin resistance tend to decrease because of reduced food intake and increased basal metabolism. The MCD model thus reflects a different pathophysiology than human NASH with respect to metabolic syndrome.

Few reported NASH-HCC models have fully incorporated all the clinical changes associated with that disease [28–30]. In our HFCD + DEN model, tumor initiation is basically dependent on a chemical carcinogen, which is artificial compared with the above spontaneous HCC models. Thus, the HFCD + DEN model cannot assess the initiation step of NASH-HCC. However, the time to HCC development in our HFCD + DEN model is 20 weeks. This is the shortest among comparable models—the high-fat and fructose diet model, MUP-uPA transgenic mice with HFD, and MC4R knockout mice with HFD, which have 48, 32, and 48 weeks, respectively. Therefore, the HFCD + DEN model may be appropriate to assess how the NASH environment promotes HCC.

Our functional analysis extracted 13 genes from HFCD + DEN and 163 from HFD32 + DEN related to HCC. Expression of Rbbp6 in HFCD + DEN and HFD32 + DEN decreased (Fig. 7b). Rbbp6 is known to interact with MDM2, and it enhanced the affinity of MDM2 for p53, which led to the ubiquitination and degradation of p53 and repression of p53-dependent gene transcription. It would be interesting to explore in detail the differences in the cancer development mechanisms among those models. One limitation with this analysis is that the samples covered only a 4-week duration. We were thus unable to observe the long-term effects of gene expression. Further investigation is required to clarify this matter.

Insulin resistance is another essential feature of NASH, which is a hepatic manifestation of metabolic syndrome [34], and insulin resistance plays an important role in exacerbation of NASH. A number of NASH models with diabetes mellitus, such as the KK-A^y^ mouse on the MCD diet and mice fed an HFD combined with STZ, have been reported [20, 21]. The KK-A^y^ mouse is a genetically engineered diabetes mellitus model with insulin resistance, but it is unclear whether it exhibits carcinogenesis. STZ destroys the insulin-producing beta cells of the pancreas; therefore, mice receiving STZ develop type 1 diabetes mellitus owing to a lack of insulin secretion rather than through the de novo development of insulin resistance. In our study, most mice exhibited insulin resistance after 12 weeks (Table 2, Fig. 3).

Although a few mice in our model developed liver cirrhosis, we believe this model will be beneficial for studying NASH-HCC. Clinically, it is unclear whether cirrhosis is a prerequisite for the development of HCC in patients with NASH. Cirrhosis and advanced fibrosis appear to be the predominant risk factors for HCC development; however, 28 % of NASH-associated HCC cases have less advanced forms of fibrosis (stage 1 or 2), and fibrosis is more frequent in men [7].

Based on CT findings, all mice in the HFCD + DEN group, but no animals in the HFCD group, had liver tumors at 20 weeks (Table 4). Histologically, focal nodular hyperplasia and dysplasia were also present. Consistent with these findings, our immunohistochemistry results showed both GS-positive HCC (Fig. 6g) and GS-negative tumors.

Leptin is a peptide hormone produced by adipocytes and is involved in appetite control and energy expenditure [35]. Obese patients often have leptin resistance and high serum levels of leptin, which exacerbate obesity and hypertension owing to a sustained increase in sympathetic nerve activity. Hyperleptinemia is also related to hyper-responsiveness to low-dose endotoxin, which is associated with NASH progression [36]. The mice in our model developed hyperleptinemia (Fig. 2e) and hypoadiponectinemia (Fig. 2f) after 20 weeks. Park et al. have reported that feeding HFD to DEN-treated mice promotes HCC development through TNF and IL-6 expression [37]. Serum levels of TNF-alpha were higher in the HFCD and HFCD + DEN groups than in the MF group at 4 and 8 weeks; however, this difference was not significant owing to large individual differences within each group (Table 1). Our findings appear to be consistent with those of another report, which found the adipocytokine level to be highest at 8 weeks and gradually decreased thereafter [21]. Although, the reason for this is unknown, serum levels of TNF-alpha and IL-6 after 12 weeks were not particularly high compared with those in the MF group.

Wolf et al. recently reported that long-term feeding an HFCD diet to mice can induce HCC, which depends on intrahepatic CD8+ T cells and NKT cells [38]. In our microarray analysis results, many genes known to affect the immune response exceeded the twofold cutoff.

A mouse model that bears the same clinical features as human NASH-HCC can be used for research into the treatment and chemoprevention of HCC resulting from NASH. One advantage of a mouse model is that a smaller drug amount is needed than with a rat model. Mice also have shorter generation times than rats. They are thus an efficient model for appropriate research by drug therapies. The most important advantage of a murine model is the anatomical and genetic similarity of mice to humans.

## Conclusion

The NASH-HCC model we describe here is simple to establish, results in rapid tumor formation, and recapitulates most of the key features of NASH. It could therefore facilitate further studies into the development, oncogenic potential, diagnosis, and treatment of this condition.

## Abbreviations

ALT, alanine aminotransferase; CRP, C-reactive protein; CT, computed tomography; DEN, diethylnitrosamine; FBS, fasting blood sugar; GS, glutamine synthetase; HCC, hepatocellular carcinoma; HFCD, high-fat, choline-deficient diet; HFD, high-fat diet; IL, interleukin; i.p, intraperitoneal; MCD, methionine- and choline-deficient diet; MF, standard diet; NAFLD, nonalcoholic fatty liver disease; NAS, NAFLD activity score; NASH, nonalcoholic steatohepatitis; SAA, serum amyloid A; TG, triglyceride; TNF, tumor necrosis factor.

## Additional file

Additional file 1: Gene symbol, probe name and data form microarray analysis, and probe name and sequence for microarray analysis. (DOCX 20 kb)
