# Supplementary material for: Development of a novel mouse model of hepatocellular carcinoma with nonalcoholic steatohepatitis using a high-fat, choline-deficient diet and intraperitoneal injection of diethylnitrosamine
Source: BMC Gastroenterol. 2016 Jun 13;16:61. doi: 10.1186/s12876-016-0477-5 (PMC4906823; doi:10.1186/s12876-016-0477-5)
Supplement: Additional file 1: — Gene symbol, probe name and data form microarray analysis, and probe name and sequence for microarray analysis. (DOCX 20 kb) [file 12876_2016_477_MOESM1_ESM.docx]

**Supplemental data 1**

Gene symbol, probe name and data form microarray analysis

| GeneSymbol | ProbeName | [HFCD+DEN] | [HFD32+DEN] | [MF] |
| --- | --- | --- | --- | --- |
| Hist2h3c2 | A_66_P127991 | 1.3311843 | 2.4704578 | -0.04374218 |
| Rbbp6 | A_55_P2175206 | 0.18262663 | 0.17233896 | -0.1008695 |
| Rbbp6 | A_55_P2788708 | -0.055692863 | -0.7708899 | -0.038912456 |
| Rbbp6 | A_55_P2737344 | -0.31242046 | -1.5118681 | -0.03502369 |
| Mterf2 | A_51_P202623 | 1.6373333 | 1.8871086 | -0.001662095 |
| Asap2 | A_52_P536731 | 1.0400697 | 1.5377203 | 0.020565351 |
| Asap2 | A_66_P111050 | 0.16805115 | 0.5825259 | -0.18093252 |
| Hist2h3c1 | A_66_P125758 | 1.1858995 | 1.9416745 | -0.006023725 |
| Rbbp6 | A_51_P285413 | 0.8101405 | 1.1833622 | -0.21191804 |
| Hist2h3c2 | A_55_P2142251 | 1.4250954 | 2.3441517 | -0.017203012 |
| Rbbp6 | A_55_P2620698 | -0.22852631 | -0.93401927 | -0.007124106 |
| Asap2 | A_55_P1957918 | 0.93321335 | 2.2784364 | 0.055792492 |
| Hr | A_55_P1958532 | 2.0229821 | 2.5426426 | 0.03350115 |

Probe name and sequence for microarray analysis

| ProbeName | Sequence |
| --- | --- |
| A_66_P127991 | CTACATAATGGTTGCTTTCAAGGAAGGTTTGAATGCCCTTAGTGTAAGTTGTTTAACGTT |
| A_55_P2175206 | GATGGGAACTACAAAAGCAGTATGTAAAAACACAATCACCCTTTTTCTACACAATTGCTT |
| A_55_P2788708 | TAAAGAAGCAGATTATGGGGAGAGAAAAGCTGAAAGCTGCCGATAGCGATCTGCAGATCA |
| A_55_P2737344 | CAGCCAGTAAGGACGAGAAGGCCAAGAAGCCTGAGAAAAATAAACTACTTGATAGCAAGG |
| A_51_P202623 | TACTGAGATTGAGTTGTGTTCAAGTTACCTCTTAATTGGTAGTTTCTGACTCATTTGCTG |
| A_52_P536731 | CAACTGTGTATGCAGAGATGATCCAGACTTAGGAAGCGCGTTTCTGAAGTTCTCTGTGTT |
| A_66_P111050 | ACAGCCTACAGCTTACATCAACCTCAGGGAAACAAGGAGCATGGAACAGAGAGGAACGGG |
| A_66_P125758 | CTATTCTTCTGTCCAGACTCTTTCTTGCCTTAGTTAACACCCTCAACAACCCTGGTCTAA |
| A_51_P285413 | GTTTAAAAAGTCAGTGTTTTTGGAGTTTTCCCCACTTTTGGATAAATGAGTTTTTTGGGG |
| A_55_P2142251 | AGCTGAAGGCTGAGATCTAGCTTACAGCTCATAATTACATCACCAGACTTCTGGAAAAAA |
| A_55_P2620698 | GTCCACCACCTCCATCTTACACCTGCTTTCGTTGTGGTAAACCTGGTCATTATATTAAGA |
| A_55_P1957918 | CAGCGCAACTGTATATACTGTATAACCTAAACGGATTATTTTCATTGTCTTAATGCAGTG |
| A_55_P1958532 | GCACACGTGCGCCAGCTCTTGTTATACTTAACAGTTCCTGCTGTGGTTTGGGGATTTTTT |
